# Assessment of the Psychological Burden Among Family Caregivers of People Living With Dementia, Parkinson's, and Alzheimer's Disease Using the Zarit Burden Interview From Bangladesh

**DOI:** 10.1002/brb3.70527

**Published:** 2025-05-07

**Authors:** Md. Rashidur Rahman, Noorjahan Aktar, Farhana Islam

**Affiliations:** ^1^ Department of Pharmacy Jashore University of Science and Technology Jashore Bangladesh

**Keywords:** Alzheimer's disease, Bangladesh, family caregivers, Parkinson's disease, Zarit Burden Interview

## Abstract

**Objective:**

This study aimed to explore the burden experienced by family caregivers of individuals with dementia, Alzheimer's disease (AD), and Parkinson's disease (PD) in Bangladesh. It sought to identify the key contributing factors and determine the primary predictors influencing caregiver burden in this context.

**Methods:**

Seventy‐five caregivers were selected on the basis of specific criteria. The Zarit burden interview was used to assess the caregiver burden among patients with dementia, AD and PD.

**Results:**

A total of 75 participants were assessed, with 50 (66.67%) being female. Most caregivers (40.0%) were spouses. Overall, caregivers experienced a moderate level of burden, with female caregivers reporting a higher burden compared to their male counterparts. Caregiver burden was significantly associated with age and the patient‐caregiver relationship.

**Conclusions:**

Greater research focus is needed on caregivers of patients with neurodegenerative diseases in developing countries.

## Graphical abstract

As indicated by Zarit Burden Interview (ZBI) scores, the majority of caregivers experienced moderate to severe burden, according to the study. This study highlights the burden experienced by caregivers of dementia, Alzheimer's disease, and Parkinson's disease patients in Bangladesh and recommends community interventions to address this issue and inform targeted interventions to reduce caregiver burden.

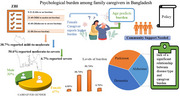

## 1 Introduction

With an aging population, dementia has become an increasingly pressing global health challenge (Wu et al. 2017). Alzheimer's disease (AD) is the most common cause of dementia, responsible for 60%–80% of all cases (Alzheimer's Association 2023). Parkinson's disease (PD) is the second most common neurodegenerative disorder after AD and is anticipated to place an increasing social and economic strain on aging populations (Geerlings et al. 2023). Dementia is a syndrome resulting from many illnesses that progressively destroy nerve cells and impair brain function, often leading to cognitive loss that surpasses normal aging (World Health Organization 2023). AD is a degenerative brain disorder that progressively impairs memory and cognitive abilities, resulting in a growing dependence on others as the condition advances (Muñoz‐Bermejo et al. 2025). PD is a chronic neurodegenerative disorder characterized by the progressive loss of dopaminergic neurons, leading to a decline in dopamine levels and causing both motor and non‐motor impairments (Wu et al. 2025). Bangladesh, home to over 2.2% of the global population, ranks as the 10th most populous country. According to the final findings of Bangladesh's 2022 Census, ongoing demographic changes have altered the country's age structure, with people aged 60 and above now representing 3.39% of its population of over 165 million (Bangladesh Bureau of Statistics 2023). The elderly are increasingly becoming a significant segment of the Bangladeshi population, and geriatric health concerns are gaining greater importance than ever before (Barikdar et al. 2016). In Bangladesh, dementia affects 1 in 12 senior people, and it is more common in women than in men (Tawsia Tajmim 2023). Awareness about AD is still in its early stages, causing patients and their families to face ongoing challenges (Rahman et al. 2017). The prevalence of PD varies globally. Although it is often associated with higher‐income countries, it is increasingly acknowledged as a major health concern in lower‐income nations (Pereira et al. 2024). In Bangladeshi culture, strong values of togetherness and empathy shape society, making it a shared responsibility for families to care for elderly members who are unwell. Bangladesh has limited specialized facilities for dementia, Alzheimer's, and Parkinson's, with the absence of social security placing the burden of care entirely on families. Caring for a loved one significantly impacts the mental well‐being of family caregivers. As the disease progresses in severity, patients require increased assistance from caregivers to maintain their quality of life, which further exacerbates the socio‐economic burden (Rahman et al. 2023). Unlike in developed countries, the limited availability of memory clinics and Alzheimer's specialists poses a serious challenge, leaving family caregivers with minimal support, resources, or guidance. Family members are often unprepared for the profound changes and challenges that come with becoming a caregiver (Whitlatch CJ and Orsulic‐Jeras 2018). Caregiver burden encompasses the challenges, difficulties, and adverse impacts experienced by individuals caring for patients (Platt 1985). It measures how caregiving affects caregivers' emotional and physical well‐being, social activities, and financial stability. A recent study (Naheed et al. 2023) revealed that the prevalence of dementia differs on the basis of significant sociodemographic factors such as age, sex, education, and marital status. Most existing literature on dementia, Alzheimer's, and Parkinson's care is from Western countries, with limited representation of Asian perspectives. The Zarit Burden Interview (ZBI) is a widely utilized tool in international research for assessing the level of burden experienced by caregivers (Park et al. 2015). In Bangladesh, no studies used this tool to assess the burden of family caregivers and compared the burden of dementia caregivers with those of other diseases. In this study, we compared the disease burden on caregivers. The aim of the study was to explore the various aspects of caregiver burden in relation to key factors and identify the primary predictors of the overall burden experienced by caregivers of individuals with dementia, AD, and PD in Bangladesh.

## 2 Materials and Methods

### 2.1 Study Site

A quantitative, descriptive, cross‐sectional study was conducted between March and September 2023 in several institutions, including the National Institute of Neuroscience & Hospital, Dhaka Medical College Hospital, Bangladesh Association for the Aged and Institute of Geriatric Medicine (BAAIGM), Dementia Day Care Centre, Jashore 250 Bed General Hospital, and other hospitals, as well as old homes in the city of Dhaka, Bangladesh. Convenience sampling was used to select participants from these settings.

### 2.2 Study Participants

Seventy‐five caregivers were selected on the basis of specific criteria. Caregivers were included in this study if they met the following criteria: (1) family caregivers who lived with the patient and were at least 18 years old, (2) caregivers who could read and comprehend the measurement tools and informed consent form, and (3) competent, reliable, and healthy caregivers of patients with a confirmed diagnosis. Caregivers were excluded if they received financial compensation for the care of the patient. This decision was made to reduce bias, as professional caregivers may have received formal training that helps them manage caregiving stress differently than family caregivers. The study excluded participants if the patient, caregiver, or any family member had a significant physical or psychiatric illness. We also excluded the absence of an identified caregiver and inability to understand self‐assessment questionnaires.

### 2.3 Study Procedure

Participants who met the selection criteria were provided with detailed information about the study and were asked to provide their consent to participate. Each participant was notified of the purpose of the study, the confidentiality and anonymity of the data, and gave consent after being informed of these details. If the patient was accompanied by more than one caregiver, the primary caregiver, who provides daily care and support, makes treatment decisions, and administers medications, was included. Participants were only enrolled in the study after receiving written informed consent. After obtaining participant consent, the project was explained individually, and a follow‐up appointment was scheduled at a convenient location. During the appointment, study objectives were explained, demographic information was recorded, and the ZBI questionnaire was administered. The study was carried out with full respect for the ethical standards outlined in the Declaration of Helsinki, ensuring the protection and well‐being of all human participants involved.

### 2.4 Data Collection

The ZBI was used to assess the caregiver burden among patients with dementia, AD, and PD. It includes 22 items that were used to assess the burden of the caregiver during the care of the client. The ZBI is a tool for measuring the burden of caregiving borne by primary caregivers. The interview contains 22 items, each of which is rated on a 5‐point Likert scale spanning from 0 (never) to 4 (nearly always) (Zarit et al. 1980). The scale was translated into a language they understood, and the subjects were given adequate time to understand the questionnaire and encouraged to ask questions for any doubts regarding the study or questionnaire. A data sheet was developed to record sociodemographic variables, which include age, gender, and relationship status. The data were collected through face‐to‐face interviews and an online form, making it easier to create and use surveys.

### 2.5 Statistical Analysis

Complete data were available for all the variables measured. Both the patient and the caregiver variables were analyzed using descriptive statistics. Pearson's correlation was used to assess the relationship between burden and its various areas with continuous variables, and Spearman's correlation when the normality assumption is violated and to comparing categorical variables an independent samples *t*‐test and Chi‐square test was used. A stepwise multiple regression analysis was conducted, with the Zarit total score as the dependent variable. Patient age and caregiver age were taken as independent variables. Multiple regressions were used to assess the ability of two control measures (patient age and caregiver age) to predict levels of burden (Zarit Burden Scale). All analyses were conducted using SPSS statistics software, version 29.0. For all statistical tests, *p *< 0.05 was considered significant.

## 3 Results

### 3.1 Characteristics of Caregivers and Patients

The study gathered sociodemographic data from the 75 caregivers. The majority of caregivers were female (66.7%) and between the ages of 46 and 55 (42.7%). There were 50 women (66.7%) and 25 men (33.3%) among the caregivers. The majority of caregivers were spouses (40.0%). In both the groups, female caregivers were more than the male caregivers. In terms of gender of patients, 34.7% of patients were female and 65.3% were male. The majority of patients were diagnosed with dementia (40.0%). Sociodemographic profile of caregivers and patients are shown in Table 1.

**TABLE 1:** Sociodemographic profile of caregivers and patients.

Characteristics of caregivers		*N* (%)
Gender	Female	66.7 (50)
	Male	33.3 (25)
Age	25–35	30.7 (23)
	36–45	26.7 (20)
	46–55	42.7 (32)
Relation with patients	Spouse	40.0 (30)
	Daughter	24.0 (18)
	Son	36.0 (27)
Characteristics of patients		
Gender	Female	34.7 (26)
	Male	65.3 (49)
Disease type	Dementia	40.0 (30)
	Alzheimer	30.7 (23)
	Parkinson	29.3 (22)

### 3.2 Levels of Burden and Its Areas

On the basis of ZBI scores, the caregiver burden was analyzed. Only 4.0% of the 75 respondents reported little to no burden, 38.7% reported mild‐to‐moderate burden, 50.6% reported moderate‐to‐severe burden, and 6.7% reported severe burden. The majority of caregivers experience a moderate‐to‐severe burden.

### 3.3 Relationship of Burden and Care Sample Characteristics

#### 3.3.1 Burden and Patient Characteristics

Correlation of burden with patient's age is shown in Table 2. To assess the size and direction of the linear relationship between the scores of Zarit burden with the patient's age, a bivariate Pearson's product‐moment correlation coefficient (*r*) was calculated. Patient's age and burden were significantly correlated with burden score (*p* = 0.01), meaning with increase in the age of patients led to an increase in the burden of care, significantly. A chi‐square test for independence with *α* = 0.05 was used to assess whether the Zarit burden level was related to disease types. The pearson chi‐square value is 4.777^a^ and *p* = 0.5. As the *p* value is *p* > 0.05, no relationship between caregiver burden with disease type of patients. The association of caregiver burden with disease conditions of patients is shown by pearson chi‐square value in Table 2.

**TABLE 2:** Burden in relation to patient age (*n* = 75).

Correlations	Zarit Burden's total score	
	Pearson correlation	*p*
Patient age	0.397^**^	0.010*
Chi‐square tests		
Pearson Chi‐square	Value	*p*
	4.777^a^	0.573

*Note*: Statistical analysis by Pearson's correlation, Chi‐Square Tests.

*p < 0.05.

#### 3.3.2 Burden and Caregiver Characteristics

An independent samples *t*‐test was used to compare the mean Zarit Burden total score of females (*n* = 50) and males (*n* = 25) caregivers. The *t*‐test was statistically significant, with mean Zarit total score of female (*M* = 44.76, SD = 11.25) was significantly higher than the male (*M* = 43.48, SD = 11.22), *t* (75) = 4.65, *p *< 0.001, two‐tailed. Mean difference of Zarit burden total scores between male and female caregivers shown in Table 3. Spearman's correlation (instead of Pearson correlation) is done because the normality assumption is violated. Spearman's rank order correlation was used to explore the relationship between the scores of Zarit burden and caregiver age. The rank order correlation was found positive but weak. The results indicate that there is correlation between the scores of Zarit burden with caregiver age. (*r_s_* = 0.281*; *p* = 0.014). Table 4 shows the correlation between caregiver burden and the caregiver's age.

**TABLE 3:** Comparing burden in relation to caregiver's gender.

Zarit Burden total score	Sex	*N*	Mean	Std. deviation	*t*
	Female	50	44.76	11.25	4.65^*^
	Male	25	43.48	11.22	

*p < 0.001.

**TABLE 4:** Relation of burden with caregiver's age.

Correlations	Zarit Burden's total score	
	Spearman's Rho correlation	*p* value
Caregiver age	0.281^a^	0.014

^a^Correlation is significant at the 0.05 level (2‐tailed).

### 3.4 Predictors of Burden by Multiple Regression Analysis

Multiple linear regression (MLR) analysis was used to examine the factors associated with caregiver burden, with the ZBI score as the dependent variable. The analysis also assessed the ability of two control measures—patient age and caregiver age—to predict levels of burden as measured by the Zarit Burden Scale, Multiple regression analysis shown in Table 5. In combination, patient age and caregiver age 11.3% of the variability in Zarit burden, *R* ^2^ = 0.113, adjusted *R* ^2^ = 0.088, *F* (2,72) = 4.572, *p *< 0.05, with the patient age recording a higher beta value (beta = 0.250, *p *< 0.05) than the caregiver age (beta = 0.163, *p *< 0.05). The F‐Statistics of 4.572 is in a significant level. Since p< 0.05, the multiple regression model is statistically significant. The analysis indicates that both patient age and caregiver age have statistically significant effects on Zarit Burden. Patient age has a stronger impact, suggesting that as patients get older, their burden tends to increase more than the impact of caregiver age. However, it's important to note that these variables only explain a relatively small portion (11.3%) of the variability in burden, indicating that other factors not included in the analysis may also play a significant role in determining burden levels.

**TABLE 5:** Multiple regression analysis.

Model		Sum of squares	df	Mean square	*F*	Sig.
1	Regression	1041.083	2	520.542	4.572	0.014b
	Residual	8197.263	72	113.851		
	Total	9238.347	74			

^a^Dependent Variable: Zarit total score.

^b^Predictors: (Constant), Caregiver age, Patient age.

## 4 Discussion

This paper explores the burden of dementia, AD, and PD caregiving within the Bangladeshi context, emphasizing the importance of culturally sensitive assessment tools. In Bangladeshi society, family bonds and community interdependence are fundamental, with a strong sense of duty often guiding caregiving roles. Similar to other South Asian cultures, including India, the commitment to caring for elderly family members with dementia frequently stems from deep‐rooted filial obligations (Kim et al. 2002). In this study, all patients with dementia, Alzheimer's, and Parkinson's were cared for by their immediate family members, in line with the cultural norm in Bangladesh, where caregiving is primarily considered a family responsibility. As indicated by their ZBI scores, the majority of caregivers experienced moderate to severe burden, according to the study. Available literature on dementia, AD, and PD caregiving reveals that the majority of caregivers endure a moderate or severe level of burden (Pattanayak et al. 2010; Vérez Cotelo et al. 2015; Yıldızhan et al. 2019). In this study, participants’ sociodemographic information was analyzed. Similar to findings from previous research in other countries, this study observed that the primary caregivers within families are typically women (Geerlings et al. 2023; Muangpaisan et al. 2010; Yin et al. 2021). An interesting finding in this study is that the majority of caregivers were spouses (40.0%), which is reflective of the strong familial bonds and the cultural expectation in Bangladesh that spouses provide primary care for one another in times of illness. In Bangladeshi society, family members, particularly spouses, are often seen as the primary source of emotional and physical support, especially when it comes to caregiving for chronic conditions like dementia, Alzheimer's, and Parkinson's. Earlier research (González‐Salvador MT et al. 1999; Nagatomo et al. 1999; Papastavrou et al. 2007) found similar patterns, suggesting the need for further research on how a patient's gender affects caregiver burden. Our study, along with several others (Akpınar et al. 2011; Almberg et al. 1998; Razali et al. 2011; Rinaldi et al. 2005), revealed that female caregivers tend to experience a higher level of burden. Both patient age and caregiver age have statistically significant effects on caregiver burden. Consistent with findings from other studies (Lim et al. 2008), this research shows that older caregivers experience higher levels of burden, largely due to the added stress of balancing multiple responsibilities. Patient age has a stronger impact, suggesting that as patients get older, caregiver burden tends to increase, and the findings of this study aligned with previous evidence (Yang et al. 2012). A similar finding was observed (Svendsboe et al. 2016; Zweig and Galvin 2014) when ranking caregiver burden from highest to lowest across each patient disease category, dementia caregivers reported the highest levels of moderate to severe burden, followed by those experiencing mild to moderate burden. The study found no significant relationship between the type of disease affecting the patient and the level of caregiver burden experienced, which aligns with similar findings from other research (Rigby et al. 2021). This means that caregivers’ perceived burden did not vary significantly whether they were caring for patients with different conditions, such as dementia, AD, or PD. The lack of a significant relationship between disease type and caregiver burden suggests that caregiver challenges are driven more by shared demands, such as cognitive and physical decline, than by the specific diagnosis. Factors like emotional resilience, social support, and coping strategies play a more crucial role in determining burden, highlighting that caregiver stress is shaped more by the overall caregiving experience than by the specific neurodegenerative disease. In this study, the ages of both the patient and caregiver were identified as significant predictors of overall caregiver burden, accounting for 11.3% of its variance. Although this model explains less than 20% of the total variance, it is still considered significant and acceptable. Caregivers face a tremendous emotional and physical toll due to the lack of effective treatments for conditions like AD. If natural products lead to safer, more effective therapies, they could delay disease progression, reduce care demands, and, consequently, lower the caregiver burden scores on the Zarit scale (Akter et al. 2021).

### 4.1 Limitations

Our study's limited sample size is one of its limitations, which could limit how broadly the results can be applied. Furthermore, relationships between different aspects of caregiver load and more objective health outcomes, like mortality and the onset of illness, are not examined in this study. Despite these limitations, the study provides valuable insights into the burden experienced by family caregivers of individuals with dementia, AD, and PD in Bangladesh.

## 5 Conclusion

This study highlights the significant emotional and physical burdens faced by family caregivers of individuals with dementia, AD, and PD in Bangladesh. It emphasizes the critical need for more tailored and effective support systems to assist caregivers in managing these challenges. Future research should aim to include larger, more diverse populations and examine the long‐term progression of caregiver burden. It would also be beneficial to explore the impact of caregiver stress on their health outcomes and investigate the potential role of natural therapies in mitigating caregiver strain. Additionally, there is a clear need for the development of community‐based support networks, respite care services, and caregiver education programs specifically designed to address the unique needs of caregivers in Bangladesh and similar cultural contexts.

## Author Contributions

**Md. Rashidur Rahman**: conceptualization, methodology, validation, investigation, writing – original draft, writing – review and editing, supervision, project administration. **Noorjahan Aktar**: methodology, validation, formal analysis, investigation, writing – original draft, writing – review and editing. **Farhana Islam**: writing – review and editing. All authors have read and agreed to the published version of the manuscript.

## Conflicts of Interest

The authors declare no conflicts of interest.

### Peer Review

The peer review history for this article is available at https://publons.com/publon/10.1002/brb3.70527.

## Data Availability

The data that support the findings of this study is available from the corresponding author upon reasonable request.
